# Thermodynamic and molecular dynamic insights into how fusion influences peptide-tag recognition of an antibody

**DOI:** 10.1038/s41598-024-59355-2

**Published:** 2024-04-15

**Authors:** Kazuhiro Miyanabe, Takefumi Yamashita, Kouhei Tsumoto

**Affiliations:** 1 Department of Chemistry and Biotechnology, School of Engineering, The University of Tokyo, Hongo 7-3-1, Bunkyo-ku, Tokyo, 113-0033 Japan; 2 Laboratory for Systems Biology and Medicine, Research Center for Advanced Science and Technology, The University of Tokyo, 4-6-1 Komaba, Meguro-ku, Tokyo, 153-8904 Japan; 3 Department of Physical Chemistry, School of Pharmacy and Pharmaceutical Sciences, Hoshi University, 2-4-41 Ebara, Shinagawa-ku, Tokyo, 142-8501 Japan; 4 Department of Bioengineering, School of Engineering, The University of Tokyo, Hongo 7-3-1, Bunkyo-ku, Tokyo, 113-0033 Japan; 5 Medical Proteomics Laboratory, The Institute of Medical Science, The University of Tokyo, 4-6-1 Shirokanedai, Minato-ku, Tokyo, 108-8639 Japan

**Keywords:** Biotechnology, Computational biology and bioinformatics

## Abstract

To understand the effect of protein fusion on the recognition of a peptide-tag by an antibody, we fused a CCR5-derived peptide-tag (pep1) to GFP and investigated its recognition by an anti-pep1 antibody, 4B08. First, to characterize the thermodynamic properties associated with the pep1-4B08 binding, isothermal titration calorimetry experiments were conducted. It was found that pep1 fused to the C-terminus of GFP (GFP-CT) enhanced the enthalpic gain by 2.1 kcal mol^−1^ and the entropic loss only by 0.9 kcal mol^−1^, resulting in an 8-fold increase in the binding affinity compared to the unfused pep1. On the other hand, pep1 fused to the N-terminus of GFP (GFP-NT) enhanced the enthalpic gain by 3.0 kcal mol^−1^ and the entropic loss by 3.2 kcal mol^−1^, leading to no significant enhancement of the binding affinity. To gain deeper insights, molecular dynamics simulations of GFP-NT, GFP-CT, and pep1 were performed. The results showed that the location of the fusion point sensitively affects the interaction energy, the solvent accessible surface area, and the fluctuation of pep1 in the unbound state, which explains the difference in the experimental thermodynamic properties.

## Introduction

Fusion of peptide-tag is common in the field of molecular biology. For example, the hexa-histidine tag and FLAG tag are frequently used to detect or purify a target protein from the cell lysate^1^. To obtain antibodies that specifically bind to a target protein efficiently, a peptide derived from the protein is fused to a target protein (e.g., bovine serum albumin or keyhole limpet hemocyanin), which enhances the immune response^2^. Traditionally, the peptide-protein fusion has been applied to the peptide therapeutics to extend the short lifetime of the peptide drug or to enhance the specificity of the target-cell recognition^3^.

To apply the fusion technique to these applications successfully, a critical factor is the interaction between the protein-fused peptide and its binding partner^4,5^. The sensitivity of detection, yield of purification, recognition by immune system, or efficacy of the therapeutics are largely dependent on the binding affinity between the peptide-tag and its binding partner. If the fusion significantly impairs the recognition of the peptide-tag by its binding partner at the molecular level, applications such as detection, purification, immunization, and therapy using the fused peptide will unexpectedly fail. Thus, it is important to investigate and to deeply understand the effect of the fusion on the binding affinity between the peptide-tag and its binding partner.

When the fusion fails due to the deterioration of the recognition of peptide-tag by the binding partner, one may consider the different construct of protein-fused peptide. In fact, it is known that the form of fusion (the selection of N-terminal fusion, C-terminal fusion, and the others) can affect the recognition significantly^1,6–9^. Another prescription might be modification of the peptide-tag to increase the binding affinity^10,11^, however it is very difficult and time-consuming. In addition, the modification cannot be used for the immunization since the antibody should be raised against original sequence of proteins.

Although the selection of the construct design is critical for the success of the fusion technology, the clear understanding of the fusion effect on the binding affinity has not yet been obtained at the molecular level. Thus, the main purpose of this study is to clarify the mechanism by which the fusion influences the interaction between the peptide-tag and binding partner. To investigate the fusion effect, we focused on the fusion of the pep1 peptide, of which sequence is DINYYTSEP, and green fluorescent protein (GFP), which is a popular molecular marker emitting a green fluorescence^12,13^. The pep1 is derived from the N-terminal region of human C-C chemokine receptor type 5 (CCR5), which plays an important role in human immunodeficiency virus (HIV) infection and can be regarded as a therapeutic target^14,15^. The pep1 peptide is recognized by its antibody, 4B08, which we prepared previously^16^. The recognition of pep1 by 4B08 was characterized in detail from the structural and thermodynamic viewpoints^16–18^. GFP has been widely used as a tool for imaging and monitoring biological processes in living cells and organisms^19^. Also, GFP is often used as a fusion partner for low-expressed or insoluble peptides due to its high solubility and stability in aqueous solution^20,21^.

To elucidate the importance of the construct design, we investigated two constructs of pep1-GFP fusion protein, GFP-NT and GFP-CT; the pep1 peptide is fused to N-terminus of GFP and C-terminus of GFP with G4S-linker in GFP-NT and GFP-CT, respectively. Below, we first characterize the thermodynamic properties of the recognition of GFP-fused pep1 by 4B08 using the isothermal titration calorimetry (ITC) method. Then, we conducted molecular dynamics (MD) simulation to explain how the fusion construct design influences the peptide-antibody interaction.

## Results and discussion

### Thermodynamic analysis of the interaction of GFP-fused pep1 with 4B08

**Figure 1:**
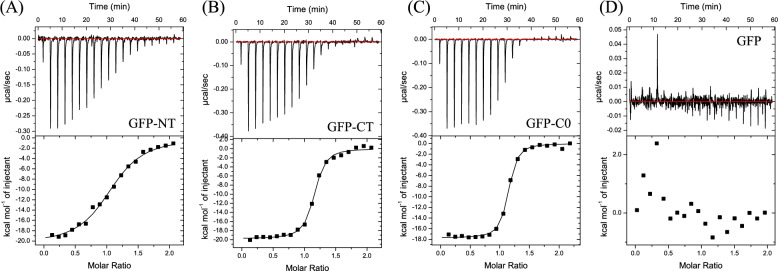
Characterization of binding to 4B08 of (**A**) GFP-NT, (**B**) GFP-CT, (**C**) GFP-C0, and GFP. The antibody was titrated with antigen at 25 °C in the present ITC experiments. In each panel, the upper plot corresponds to the titration kinetics, whereas the lower plot represents the integrated binding isotherms. Molar ratio refers to the relative concentration of peptide-to-antibody in the cell. The binding enthalpy (*ΔH*) and the dissociation constant (*K*_D_) were obtained by non-linear regression of the integrated data to a one-site binding model with the program ORIGIN. The results are given in Table 1.

To investigate the effect of protein fusion on the interaction between a peptide and its antibody, we employed the CCR5-derived peptide, pep1, and its single-chain fragment variable (scFv)-form antibody, 4B08, as a model pair. Here, the pep1 peptide was fused to N-terminus of GFP and C-terminus of GFP via a G4S-linker in the GFP-NT and GFP-CT constructs, respectively. To quantitatively characterize how 4B08 recognizes the two constructs, the thermodynamic properties were measured by ITC experiments. Results were tabulated in Table 1. In the ITC experiment, we confirmed that the binding heat between GFP and 4B08 was undetectable (Fig. 1), which suggests that the non-specific interaction of GFP-region of GFP-fused pep1 with 4B08 is negligible.

**Table 1:** Thermodynamic parameters of binding of GFP-fused pep1 to 4B08.

	Unfused pep1^a^	GFP-NT	GFP-CT	GFP	GFP-C0
*N*	0.91 ± 0.04	1.06 ± 0.05	1.07 ± 0.03	N.D.	1.08 ± 0.01
*ΔG* (kcal mol^−1^)	− 8.8 ± 0.1	− 8.6 ± 0.1	− 9.9 ± 0.1	N.D.	− 9.9 ± 0.1
*ΔH* (kcal mol^−1^)	− 16.8 ± 0.1	− 19.8 ± 0.5	− 18.9 ± 0.3	N.D.	− 18.2 ± 0.2
− *TΔS* (kcal mol^−1^)	8.0 ± 0.2	11.2 ± 0.5	8.9 ± 0.3	N.D.	8.2 ± 0.3
*K*_D_ (nM)	370 ± 5.6	508 ± 46	50 ± 5	N.D.	51 ± 7

^a^Thermodynamic properties for the unfused pep1 were taken from Ref.^17^.

The ITC experiments clearly showed that the GFP-fusion significantly influences the binding affinity. While the binding affinity between the unfused pep1 and 4B08 was 370 ± 5.6 nM, the binding affinity were 50 ± 5 nM and 508 ± 46 nM for GFP-CT and GFP-NT, respectively. Interestingly, the N-terminal fusion and C-terminal fusion displayed different influence on the 4B08-recognition. The C-terminal fusion enhanced the 4B08 binding affinity drastically (8-fold), whereas the N-terminal fusion does not. The binding free energy (*ΔG*) was − 9.9 ± 0.1 kcal mol^−1^ for GFP-CT, which was larger than the *ΔG* for the GFP-NT (− 8.6 ± 0.1 kcal mol^−1^) and unfused pep1 (− 8.8 ± 0.1 kcal mol^−1^).

To understand the fusion effect in more detail, we decomposed the binding free energy into the enthalpic contribution (*ΔH*) and the entropic contribution (− *TΔS*). The binding enthalpy was more favorable for both of GFP-NT and GFP-CT than for the unfused pep1; *ΔH* =  − 19.8 ± 0.5 and − 18.9 ± 0.3 kcal mol^−1^ for GFP-NT and GFP-CT, while *ΔH* =  − 16.8 ± 0.1 kcal mol^−1^ for the unfused pep1. In the enthalpic aspect, the N-terminal fusion enhances the binding enthalpy more largely than the C-terminal fusion. However, in the entropic aspect, while GFP-CT possessed slightly more unfavorable change of entropy (− *TΔS* = 8.9 ± 0.3 kcal mol^−1^) than the unfused pep1 (− *TΔS* = 8.0 ± 0.2 kcal mol^−1^), the entropic change for GFP-NT (− *TΔS* = 11.2 ± 0.5 kcal mol^−1^) was more unfavorable than for the unfused pep1. Consequently, the C-terminal fusion overcomes the enthalpy–entropy compensation problem^22,23^ and enhances the binding free energy (affinity) largely, whereas the N-terminal fusion effect on the binding affinity is very small.

### Molecular dynamics (MD) simulation of GFP-fused peptide

To obtain deeper insight into the GFP-fusion effect at the molecular level, we conducted MD simulations of unfused pep1, GFP-NT and GFP-CT for 600 ns. Here, we focused on the unbound state, following the previous MD analysis method, which qualitatively explained the thermodynamic properties associated with the peptide-antibody binding^16,17^. Note that the ITC experiment also indicates that the interaction between GFP and 4B08 may be negligibly small (Fig. 1). The last 450 ns data were used for the analyses, while the first 150 ns data were used for the equilibration of the systems.

First, we calculated the total interaction energy of the pep1 part (Table 2). Note that the total interaction energy was defined as the sum of the Coulomb and Lennard–Jones (LJ) interaction energies inside the pep1, between the pep1 and solvents, and between the pep1 and GFP. While the total interaction energy for the unfused pep1 was − 1238.3 ± 0.3 kcal mol^−1^, those for GFP-CT and GFP-NT were − 1194.8 ± 1.4 kcal mol^−1^ and − 1102.2 ± 2.2 kcal mol^−1^, respectively. These results indicate that the fusion of GFP destabilizes the unbound state of the pep1, which can explain why the GFP-fusion enhances the change in enthalpy upon binding to 4B08. In addition, the total interaction energy for GFP-NT is smaller than that for GFP-CT, which is consistent with the fact that the binding enthalpy for GFP-NT is larger than that for GFP-CT.

**Table 2:** Interaction energy of the pep1 part (kcal mol^-1^).

Component		Unfused pep1	GFP-NT	GFP-CT	GFP-C0
intra-pep1	Coulomb	− 850.7 ± 0.3	− 677.5 ± 2.8	− 773.9 ± 1.7	− 771.4 ± 1.5
	LJ	− 21.9 ± 0.1	− 22.3 ± 0.4	− 22.9 ± 0.3	− 22.5 ± 0.3
pep1-solvent	Coulomb	− 339.4 ± 0.3	− 246.9 ± 3.3	− 292.1 ± 4.2	− 298.0 ± 4.9
	LJ	− 26.2 ± 0.1	− 24.4 ± 1.4	− 16.1 ± 1.2	− 20.3 ± 1.3
pep1-GFP	Coulomb	NA	− 105.1 ± 2.8	− 60.4 ± 3.3	− 72.8 ± 3.6
	LJ	NA	− 26.0 ± 2.8	− 29.4 ± 2.6	− 21.0 ± 2.8
Total interaction energy		− 1238.3 ± 0.3	− 1102.2 ± 2.2	− 1194.8 ± 1.4	− 1206.0 ± 1.5

Although the GFP-fusion introduced the pep1-GFP interaction, the intra-pep1 interaction and pep1-solvent interaction were diminished. In particular, the intra-pep1 coulomb energy was − 850.7 ± 0.3 kcal mol^−1^ for the unfused pep1, while those for GFP-NT and GFP-CT were − 677.5 ± 2.8 kcal mol^−1^ and − 773.9 ± 1.7 kcal mol^−1^, respectively. Also, the pep1-solvent coulomb energy for the unfused pep1 (− 339.4 ± 0.3 kcal mol^−1^) was significantly lower than those for GFP-NT and GFP-CT (the values were − 246.9 ± 3.3 and − 292.1 ± 4.2 kcal mol^−1^, respectively). Similar tendency can be seen in the number of hydrogen bonds (Table S1).

In contrast, it was found that the GFP-fusion increases the binding entropy (Table 1). To understand the effect on the binding entropy, we first investigated the solvent accessible surface area (SASA) of the pep1 part. As a result, the SASA for the unfused pep1 was significantly larger than those for the GFP-NT and GFP-CT (Table S1). This is simply because the GFP part contacts the pep1 and masks the pep1 surface. In general, the SASA indicates the number of contact solvents, which significantly attenuate their translational motions and contribute to the entropy reduction^17,24,25^. Thus, the decrease in the SASA of the pep1 part can enhance the entropy in the 4B08-free state; this can qualitatively explain the experimental tendency that the GFP-fusion enhanced the entropic loss upon binding 4B08 (Table 1).

**Figure 2:**
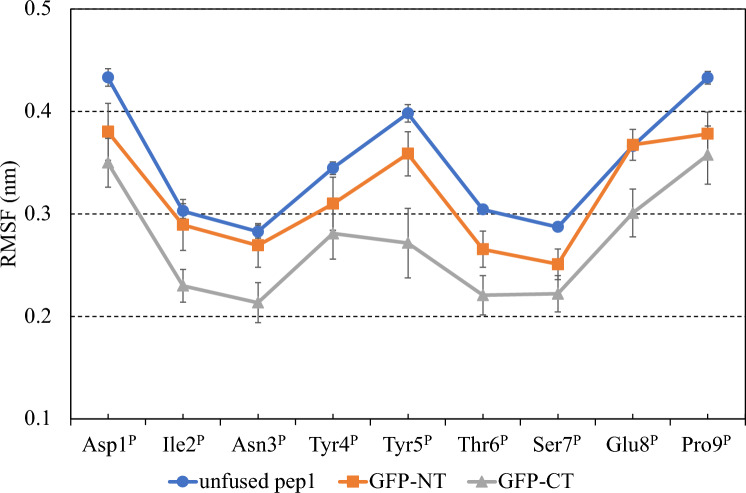
Structural fluctuation of the pep1 part.

However, the SASA analysis cannot explain why the binding entropic loss is larger for the GFP-NT than for the GFP-CT. To understand the entropic difference between GFP-NT and GFP-CT, we calculated the root mean square fluctuation (RMSF) values of the pep1 part of the GFP-NT and GFP-CT as well as the unfused pep1 (Fig. 2). In this paper, to make the comparison simple, the pep1 residues (DINYYTSEP) are numbered independently from Asp1^P^ at N-terminus to Pro9^P^ at C-terminus, where the superscript P denotes the pep1 residues. GFP-CT showed significantly lower RMSF values than unfused pep1, while the RMSF value of GFP-NT was slightly lower than that of pep1. Although the fused GFP restricted the motion of the pep1 part, the degree of restriction observed in GFP-CT was significantly more pronounced than in GFP-NT. This result indicates that the GFP-CT fusion reduces the binding entropy loss much more than the GFP-NT fusion, which might be attributed to the reduction of the flexibility of the pep1 part caused by the GFP fusion.

**Figure 3:**
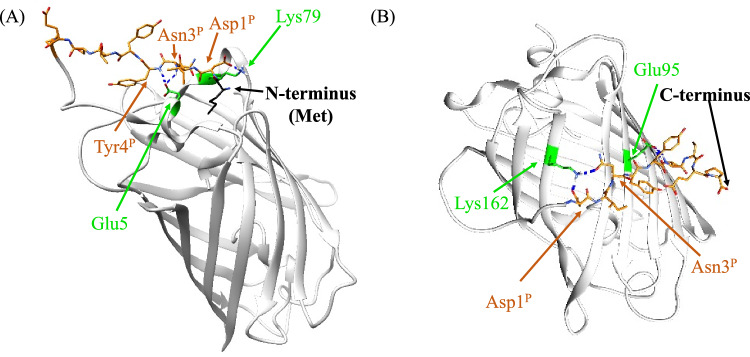
Selected snapshots of (**A**) GFP-NT and (**B**) GFP-CT observed in MD simulations. Orange sticks represent the pep1 part, while gray ribbons represent GFP. Green sticks represent GFP residues that significantly interacted with the pep1 part.

To understand the molecular basis underlying the fusion effect in more detail, we decomposed the pep1-GFP interaction energy into the contributions of GFP residues. Table 3 shows GFP residues of which interaction energies are larger than − 1.5 kcal mol^−1^; six and ten GFP residues are listed for GFP-NT and GFP-CT, respectively. As indicated by these results, we found that the pep1 parts of GFP-NT and GFP-CT interacted with different surface area of GFP (Fig. S1). GFP-CT tends to contact residues located at the side of the β-barrel (Arg73, Glu95, Lys158, Lys162, Asn164, Lys166, Arg168, and Tyr200) and disordered C-terminal chain (Gly228 and Thr230). On the other hand, GFP-NT frequently interacts with residues located at the top of the β-barrel (Lys79, Arg80) or the disordered N-terminal chain (Phe1, Ser2, Lys3 and Glu5). Figure 3 shows selected structures of the GFP-NT and GFP-CT constructs, which were typically observed in MD simulations. The side of β-barrel is solid, whereas the top of β-barrel, which consists of short α-helices and loops, is flexible. This might be a reason why the fluctuation of the pep1 part is significantly smaller in the GFP-CT construct than the GFP-NT construct (Fig. 2). Also, it was observed that the pep1 part tends to interact with basic residues of GFP for both the GFP-NT and GFP-CT systems, which might be attributed to the acidic sequence of pep1 (Asp1^P^ and Glu8^P^).

**Table 3:** GFP residues with significant interactions with the pep1 part.

GFP residue	Interaction energy (kcal mol^−1^)	Distance from Ser2 (nm)	Distance from Gly228 (nm)
GFP-NT			
Lys79	− 3.24 ± 1.41	0.89	1.27
Phe1	− 3.22 ± 0.93	N.A.	N.A.
Arg80	− 2.04 ± 0.73	0.83	1.34
Glu5	− 2.02 ± 1.27	0.58	2.03
Lys3	− 2.00 ± 0.67	0.38	2.34
Ser2	− 1.57 ± 0.49	0.00	2.10
GFP-CT			
Lys162	− 2.77 ± 1.44	2.04	1.63
Lys158	− 2.50 ± 1.29	1.95	2.35
Glu95	− 2.29 ± 1.75	2.36	2.39
Arg168	− 2.15 ± 1.86	3.43	2.13
Thr230	− 2.09 ± 1.22	N.A.	N.A.
Asn164	− 2.08 ± 1.05	2.49	1.51
Gly228	− 1.72 ± 0.95	2.10	0.00
Tyr200	− 1.68 ± 0.80	1.99	0.60
Lys166	− 1.53 ± 0.56	3.02	1.76
Arg73	− 1.52 ± 1.13	1.52	1.30

Ranking of GFP residues by interaction energy. Amino acid residues of which interaction energies are more negative than − 1.5 kcal mol^−1^ are presented. The distance from fusion point of GFP-NT (Ser2) or that of GFP-CT (Gly228) to each residue are also listed. N.A. means that the distance was not analyzed because the residue was in an unstructured domain.

The fusion effect can be considered as the effect restraining pep1 to the GFP fusion point. To see the fusion effect from the structural aspect, we focused on the distances from the fusion point to each of GFP residues that interact with the pep1 part significantly (Table 3). Here, we selected Ser2 and Gly228 as the GFP-NT fusion point and GFP-CT fusion point, respectively, and calculated the C$\alpha$-C$\alpha$ distances using the GFP structure (PDB ID: 1EMA). The results showed that the GFP-NT fusion point is closer to the six interacting GFP residues in GFP-NT than to the ten interacting GFP residues in GFP-CT (Table 3). The distances in GFP-NT ranged from 0.38 to 0.89 nm, while the distances in GFP-CT ranged from 1.42 to 3.43 nm. Since the length of the GFP-NT linker (the G4S part plus the disordered Phe1) is 2.72 nm, the pep1 part of the GFP-NT construct can access all the six interacting GFP residues in GFP-NT but not some of the ten interacting GFP residues in GFP-CT. On the other hand, the GFP-CT fusion point was at comparable distances to both the six interacting GFP residues in GFP-NT and the ten interacting GFP residues in GFP-CT; the former distances ranged from 1.27 to 2.34 nm, while the latter distances ranged from 0.60 to 2.39 nm. Since the length of the GFP-CT linker (G4S plus seven disordered GFP C-terminal residues) is 4.66 nm, the pep1 part of GFP-CT can access not only the ten interacting GFP residues in GFP-CT but also the six interacting GFP residues. However, the pep1 part of GFP-CT was observed to interact with the side of the β-barrel but not with the top, suggesting that the side has a favorable interacting area. This is essentially consistent with the fact that the pep1 part has larger interaction energy in GFP-CT than in GFP-NT (Table 2).

Given these results, the side of the β-barrel has a more favorable interacting area for the pep1 part than the top. In GFP-NT, the pep1 part interacts with the top of β-barrel but not with the side, which should be attributed to the conformational restriction introduced by the fusion. In GFP-CT, the pep1 part is structurally allowed to access the interacting area in the side and thus preferentially interacts with the side.

### Thermodynamic properties of a newly designed GFP-fused peptide: computational prediction and experimental validation

Above, MD simulations revealed how the interaction between the pep1 and GFP parts influenced the thermodynamic properties associated with the binding of pep1 to 4B08. These findings indicate the molecular basis by which the fusion point of peptide-tag and protein influences its antibody binding and provides valuable insights for the design of fusion protein constructs with improved binding characteristics. The present results indicate that the fusion effect on the unbound state qualitatively explains the fusion effect on the thermodynamic properties very well. Therefore, we consider that the fusion effect on the binding state to be minor, as reported in the previous studies^16,17^. To further examine whether the computational analysis of the unbound state can clearly predict and explain the fusion effect on the thermodynamic properties, we introduced a new GFP-fused peptide, denoted as GFP-C0. This design excludes the G4S linker from GFP-CT to alter the interactions between the pep1 and GFP parts, modulating the unbound state and thus its binding affinity.

First, we conducted MD simulations of GFP-C0 under almost the same conditions as in the GFP-CT and GFP-NT systems. As expected, the interaction area was altered by the exclusion of G4S linker (Table 3 and Table S2). For example, some of GFP-C0 residues (Lys158, Glu95, and Arg168), which interact with the pep1 part in case of GFP-CT, significantly decreased their interaction energy with the pep1. Note that these three residues are far away from the fusion point of GFP-CT than other interacting residues. This is consistent with the shorter linker of GFP-C0 compared to that of GFP-CT.

We found that the total interaction energy acting on the pep1 part in the GFP-C0 system was larger than that of GFP-NT and GFP-CT (Table 2), which indicates that the binding enthalpy for GFP-C0 should be less favorable than that of GFP-NT and GFP-CT. To validate these predictions, we additionally conducted the ITC experiment for GFP-C0. As shown in Table 1, the binding enthalpy of GFP-C0 was − 18.2 ± 0.2 kcal mol^**−**1^, which is smaller than that of GFP-NT and GFP-CT (− 19.8 ± 0.5 and − 18.9 ± 0.3 kcal mol^**−**1^, respectively). To assess the entropic contribution, we further calculated SASA (Table S1). GFP-C0 exhibited significantly larger SASA (982.4 ± 30.0 Å^2^) than that of GFP-NT and that of GFP-CT (920.8 ± 28.7 and 900.7 ± 26.0 Å^2^), suggesting that the binding entropy of GFP-C0 should be more favorable than that of GFP-NT and that of GFP-CT. This is consistent with the ITC results, which shows that the binding entropy is smaller for GFP-C0 than for GFP-CT and for GFP-NT.

## Conclusion

Fusion of a peptide-tag to a protein is a fundamental technique in the field of protein engineering, but the origin of the fusion effect has not been understood clearly. To elucidate the fusion effect in detail, we employed a CCR5-derived peptide-tag (pep1) recognized by the 4B08 antibody and fused it to GFP. The ITC experiments showed that C-terminal fusion (GFP-CT) led to an impressive eight-fold increase in binding affinity compared to the unfused pep1, while N-terminal fusion (GFP-NT) showed no significant change. These results indicate that the fusion effect is largely dependent on the design of fusion constructs. To understand why GFP-CT enhances the binding affinity, we decomposed the binding free energy (*ΔG*) into the enthalpic contribution (*ΔH*) and entropic contribution (*-TΔS*). From the enthalpic aspect, both GFP-NT and GFP-CT enhanced the binding enthalpy by 2.1 and 3.0 kcal mol^**−**1^, respectively. From the entropic aspect, whereas GFP-NT enhanced the entropic loss and compensated the enthalpic gain, the GFP-CT increased the entropic loss only by 0.9 kcal mol^**−**1^. This means that the difference in the binding affinity is primarily attributed to the entropic effect.

To gain deeper insights into the fusion effect at the molecular level, we conducted MD simulations for the GFP-NT, GFP-CT, and unfused pep1. Here, we focus on the unbound state, as the interaction between GFP and 4B08 should be so negligibly small (Fig. 1) that we can assume that the GFP-fusion does not influence the binding state. From the enthalpic aspect, we found that both GFP-NT and GFP-CT exhibited suppressed interaction energies between the pep1 region and the surrounding environment, indicating that the GFP-fusion enhances the binding enthalpy by destabilizing the unbound state of the pep1. From the entropic aspect, we found that the pep1 part of GFP-CT mainly interacts with the solid β-barrel side, whereas that of GFP-NT does with flexible helices and loops. As a result, GFP masks the solvent accessible surface of the pep1 part, which enlarges the loss of binding entropy. Also, the fluctuation of the pep1 region was significantly smaller for GFP-CT than for GFP-NT, providing an explanation for the difference in the entropic loss between the two constructs.

From the structural viewpoint, the pep1 part of GFP-NT cannot interact with that of GFP-CT, while the pep1 part of GFP-CT can interact with that of GFP-NT. The total interaction energy of the pep1 part is larger in the GFP-CT system than in the GFP-NT system, suggesting that the β-barrel side area is more favorable area for the pep1 part than the top area. Thus, the pep1 part of the GFP-CT construct mainly interacts with the β-barrel side area rather than the top area of GFP. On the other hand, the pep1 part of the GFP-NT interacts with the top area of GFP, but not with the side area. These results highlight how the location of the fusion point influences the interaction between the pep1 part and GFP part, impacting the thermodynamic properties associated with the 4B08 binding.

In this study, using thermodynamic analyses and MD simulations, we have unveiled that the structural feature of the GFP-CT construct effectively suppresses the motion of peptide, resulting in the enhancement of the binding affinity. This suggests that the MD simulation analysis can aid in the design of the highest affinity construct. Note that, although we did not analyze the structural and dynamic features of the bound state due to limited computational resources available^26^, this aspect is crucial for predicting the binding affinity more precisely. We believe that the rational design of the high-affinity fusion construct based on the accurate MD simulations can reduce the need for the experimental try-and-error procedures.

## Materials and methods

### Preparation of expression vectors

The preparation of vector for 4B08 antibody was previously described^16^. The enhanced GFP^27,28^ was used as a GFP molecule, and gene of GFP was subcloned into the expression vector pET-28(b) (Novagen). Restriction sites for the insertion at N- and C-terminus were *Eco*RI and *Xho*I, respectively. In the GFP-NT and GFP-CT constructs, the pep1 peptide was genetically fused to the N- or C-terminus of GFP, and short linker G4S were introduced between pep1 and GFP. In the GFP-C0 construct, the pep1 peptide was directly fused to the C-terminus of GFP. Furthermore, hexa-histidine tag was introduced into the terminus of GFP, to which the pep1 was not fused.

### Preparation of proteins

The preparation of 4B08 antibody was previously described^16^. For the preparation of GFP-NT, GFP-CT, and GFP-C0, *Escherichia coli* strain BL21(DE3) carrying the expression vector of protein-fused peptides was grown overnight at 28 °C, 170 rpm in LB plate medium. The cells were diluted into 100 mL of LB medium and cultured at 37 °C and 140 rpm until the OD_600_ reached a value of 0.2. At that point isopropyl β-d-1-thiogalactopyranoside was added to the cell culture to a final concentration of 0.5 mM and the cell culture was left overnight at 37 °C. Cells were harvested by centrifugation at 7,000 × *g* for 20 min at 4 °C. The cell-pellet was resuspended in 40 mL of TRIS buffer (20 mM Tris, 500 mM NaCl, pH 8.0) supplemented with 5 mM imidazole, and the cells lysed with an ultrasonic cell-disrupting UD-201 instrument (TOMY, Japan). The cell lysate was subsequently centrifuged at 4 °C, 40,000 × *g* for 30 min. The supernatant was collected, and further purification was conducted by size exclusion chromatography (AKTA purifier with Hiload 16/600 superdex 75 pg, GE healthcare) with TRIS buffer at a flow rate of 1.0 mL/min at 4 °C.

### Isothermal titration calorimetry (ITC)

The interaction between antibody and antigen was observed by the isothermal titration calorimetry (MicroCal Auto-iTC200 or iTC200, Malvern). Prepared protein-fused peptides and antibody were dialyzed overnight in PBS buffer (137 mM NaCl, 2.7 mM KCl, 10 mM Na_2_HPO_4_・12H_2_O, 1.76 mM KH_2_PO_4_, pH 7.4). The concentration of 4B08 (in the cell) and peptide (in the syringe) was 9–11 μM and 100–120 μM. Titrations were carried out at 25 °C with a reference power of 5 μcal/s, and a stirring rate of 1000 rpm. Each experiment consisted of a single 0.5-μL injection of peptide followed by 18 additional injections of 2.0 μL each with an interval between injections of 180 s. Thermodynamic parameters were calculated with the program ORIGIN 7.0 (OriginLab) using a single-site binding model. For each construct, measurement was performed four times at 25 °C.

### Molecular dynamics (MD) simulation

The GROMACS ver. 4.6.7^29^ with the CHARMM36m force field was used^30^. The initial structures of GFP-CT and GFP-NT were constructed based on the crystal structure (PDB ID: 1EMA); the missing residues were computationally generated and the F64L, F99S, M153T, and V163A mutations were introduced to completely mimic the GFP sequence used in the experiment. The extended structure of the unfused pep1 was computationally generated as an initial structure. The force field of the GFP chromophore was prepared by using the CGenFF program^31^. Each system was solvated with TIP3P water molecules under the periodic boundary condition. Na and Cl ions were added to neutralize the protein charge, then further ions were added corresponding to a salt solution of concentration 0.14 M: The GFP-CT consists of a GFP-CT construct, 59,598 water molecules, 172 Na^+^ ions, and 166 Cl^-^ ions, and the GFP-NT system consists of a GFP-NT construct, 36,807 water molecules, 109 Na^+^ ions, and 104 Cl^-^ ions, while and the unfused pep1 systems consists of a pep1 peptide, 2911 water molecules, 11 Na^+^ ions, and 9 Cl^-^ ions. The GFP-C0 consists of a GFP-C0 construct, 38,102 water molecules, 114 Na^+^ ions, and 108 Cl^−^ ions.

In this study, we conducted ten MD simulations for each system. After the energy minimization, the systems were equilibrated by 300 ps MD simulations while restraining the protein heavy atom positions. Then, removing the position restraints, we conducted the MD simulations for 600 ns. Here, the Nose–Hoover thermostat and Parrinello-Rahman barostat were used to keep the temperature and pressure constant (T = 298 K and p = 1 atm), respectively. To treat the long-ranged electrostatic interaction, the particle mesh Ewald method was used, while a cutoff distance of 10 Å for Coulomb and van der Waals interactions was used. All bond lengths were fixed with the LINCS algorithm, and the time step was set to 3 fs. The last 450 ns data were used for the energetic, number of hydrogen bond, solvent accessible surface area (SASA), and root mean square fluctuation (RMSF) analyses. The average and standard error of the analysis data were calculated over the ten MD simulations. In the present energetic analysis, we defined the interaction energy as the sum of short-ranged Coulombic interaction energy and Lennard–Jones interaction energy.

### Supplementary Information

Supplementary Information.

## Data Availability

The datasets used and/or analyzed during the current study are available from the corresponding author on reasonable request.

## Abbreviations

CCR5: C-C chemokine receptor type 5
GFP: Green fluorescent protein
ITC: Isothermal titration calorimetry
MD: Molecular dynamics
RMSF: Root mean square fluctuation
scFv: Single-chain variable fragment
SASA: Solvent accessible surface area
