# Supplementary material for: Thermodynamic and molecular dynamic insights into how fusion influences peptide-tag recognition of an antibody
Source: Sci Rep. 2024 Apr 15;14:8685. doi: 10.1038/s41598-024-59355-2 (PMC11018781; doi:10.1038/s41598-024-59355-2)
Supplement: Supplementary file 1 — Supplementary Information. [file 41598_2024_59355_MOESM1_ESM.pdf]

Supporting information

Thermodynamic and molecular dynamic insights into how fusion influences peptide-tag recognition of an antibody

Kazuhiro Miyanabe,[1] Takefumi Yamashita, [2, 3, *] and Kouhei Tsumoto [1, 4, 5, *]

[1] *Department of Chemistry and Biotechnology, School of Engineering, The University of Tokyo, Tokyo, Japan.*

[2] *Laboratory for Systems Biology and Medicine, Research Center for Advanced Science and Technology, The University of Tokyo, Tokyo, Japan.*

[3] *Department of Physical Chemistry, School of Pharmacy and Pharmaceutical Sciences, Hoshi University, Tokyo, Japan.*

[4] *Department of Bioengineering, School of Engineering, The University of Tokyo, Tokyo, Japan.*

[5] *Medical Proteomics Laboratory, The Institute of Medical Science, The University of Tokyo, Tokyo, Japan.*

Supplemental Table

Table S1: Structural characteristics of GFP-fused peptides

	unfused pep1	GFP-NT	GFP-CT	GFP-C0
number of hydrogen bonds				
intra-pep1	0.84 ± 0.02	0.72 ± 0.13	0.73 ± 0.19	0.63 ± 0.05
pep1-solvent	46.99 ± 0.05	36.80 ± 0.55	40.57 ± 0.68	41.98 ± 0.71
pep1-GFP	NA	3.13 ± 0.54	3.79 ± 0.47	3.02 ± 0.45
total	47.83 ± 0.03	40.66 ± 0.29	45.08 ± 0.38	45.63 ± 0.35
SASA of pep1 (Å^2)	1264.1 ± 1.2	920.8 ± 28.7	900.7 ± 26.0	982.4 ± 30.0

As we observed in the interaction energy, the number of hydrogen bonds formed by the pep1 portion exhibits a similar trend. In fact, the GFP-fusion reduced the total number of hydrogen bonds. The number for unfused pep1 was 47.83 ± 0.03, while those for GFP-NT, GFP-CT, and GFP-C0 were 40.66 ± 0.29, 45.08 ± 0.38, and 45.63 ± 0.35, respectively. Although additional hydrogen bonds formed between pep1 and GFP in the GFP-fused peptides, more hydrogen bonds were lost between pep1 and solvents. This change in the number of hydrogen bonds may partly explain the enhancement of the binding enthalpy caused by GFP-fusion from a structural perspective. SASA of the pep1 part for each construct was also tabulated for the discussion in the main text.

Table S2: GFP-C0 residues with significant interactions with the pep1 part

GFP residue	Interaction energy (kcal mol^{-1})	Distance from Gly228 (nm)
Lys166	−5.89 ± 1.93	1.76
Tyr151	−1.93 ± 0.72	0.97
Arg73	−1.79 ± 1.57	1.30
Tyr200	−1.55 ± 0.64	0.60
Lys162	−1.53 ± 0.81	1.63

Residues of GFP-C0 are arranged from highest interaction energy to lowest interaction energy. Amino acid residues of which interaction energies are more negative than −1.5 kcal mol^{-1} are presented. The distance between fusion point of GFP-CT (Gly228) and each residue are also indicated.

Supplemental Figure

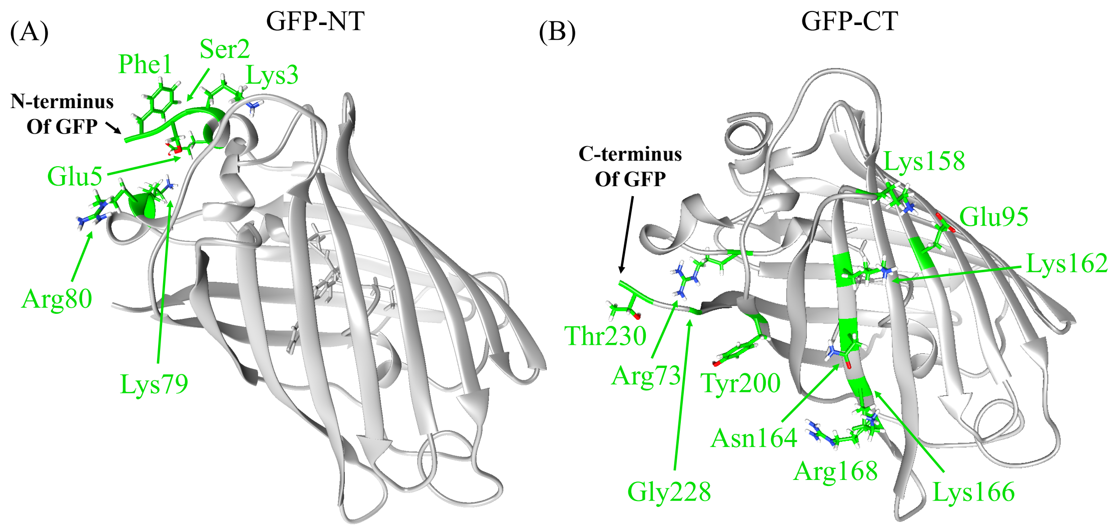

Figure S1: (A) GFP-NT and (B) GFP-CT residues of which interaction energies are more negative than −1.5 kcal mol^{-1}. Gray ribbons represent GFP. Green sticks represent GFP residues that significantly interacted with the pep1 part.
